# Surface modification of microparticles causes differential uptake responses in normal and tumoral human breast epithelial cells

**DOI:** 10.1038/srep11371

**Published:** 2015-06-12

**Authors:** Tania Patiño, Jorge Soriano, Lleonard Barrios, Elena Ibáñez, Carme Nogués

**Affiliations:** 1 Unitat de Biologia Cel·lular, Departament de Biologia Cel·lular, Fisiologia i Immunologia. Facultat de Biociències. Universitat Autònoma de Barcelona, 08139 Bellaterra, Spain

## Abstract

The use of micro- and nanodevices as multifunctional systems for biomedical applications has experienced an exponential growth during the past decades. Although a large number of studies have focused on the design and fabrication of new micro- and nanosystems capable of developing multiple functions, a deeper understanding of their interaction with cells is required. In the present study, we evaluated the effect of different microparticle surfaces on their interaction with normal and tumoral human breast epithelial cell lines. For this, AlexaFluor488 IgG functionalized polystyrene microparticles (3 μm) were coated with Polyethyleneimine (PEI) at two different molecular weights, 25 and 750 kDa. The effect of microparticle surface properties on cytotoxicity, cellular uptake and endocytic pathways were assessed for both normal and tumoral cell lines. Results showed a differential response between the two cell lines regarding uptake efficiency and mechanisms of endocytosis, highlighting the potential role of microparticle surface tunning for specific cell targeting.

In recent years, the development of micro- and nanotechnology for biomedical applications has attracted a great deal of attention. This is due to the unique and controllable physicochemical properties of micro- and nanoparticles, which allow them to integrate multiple functions such as biosensing^1^,^2^,^3^,^4^, targeting^5^,^6^,^7^,^8^ and drug delivery^9^,^10^,^11^. Recently, a wide range of studies has focused on the fabrication and characterization of new micro- and nanoparticles for biomedical purposes. However, a deeper understanding of their interactions with biological systems at the cellular level is required for the design of new multifunctional micro and nanosystems with potentiated effects on target cells and diminished side effects on healthy ones.

Different studies have revealed that size^12^,^13^,^14^,^15^, shape^16^,^17^ and surface functionalization of particles^18^ are key factors regarding their internalization by cells. Particularly, surface charge of micro- and nanoparticles has been described to play a critical role in their internalization by cells, the cationic particles being more efficiently internalized than the anionic ones^19^,^20^, in the majority of cases. Thus, with the aim to obtain cationic particles, several approaches have been performed, including their coating with cationic lipids or polymers. Specifically, particle coating with Polyethyleneimine (PEI) has been of particular relevance^21^,^22^,^23^ due to the widely known protonsponge effect, which involves the protonation of PEI amine groups that leads to endo/lysosomal pH buffering, membrane disruption, and ultimate liberation of the content to the cytosol^24^. Moreover, the proton sponge effect has been shown to be more efficiently achieved when a high molecular weight of PEI is used^25^.

On the other hand, micro- and nanoparticles cellular uptake and the mechanism of internalization can differ among cell types^12^,^13^,^18^,^26^. This, however, has received less attention in the literature.

Specific antibodies or peptides against certain cell surface markers are used to enhance particle internalization in target cells^27^,^28^,^29^,^30^. However, other intrinsic properties of non-targeted particles can also have a differential effect upon different cell lines^18^. Moreover, whilst there is a wide range of particle-cell interaction studies conducted with nanoparticles, less is known about the interaction of larger particles with cells. More research is thus required to improve the strategies for biomedical applications of micron-sized delivery systems, as they have been proved to be a good approach for drug delivery^31^,^32^,^33^,^34^,^35^.

Hence, the aim of the present work was to provide an integrated study about the impact of different non-specific surface modifications of microparticles upon their interaction with different cell types, in terms of cytotoxicity, uptake efficiency, mechanism of internalization and intracellular fate. With this purpose, 3-μm polystyrene microparticles were functionalized with a fluorescently-labeled non-specific antibody. Then, two types of PEI, both differing on their structure and molecular weight, were used for microparticle coating. The cytotoxicity and internalization of those microparticles were studied in two human breast epithelial cell lines, one normal and another tumoral.

## Results

### Effect of 0.05 mM PEI on cell viability

**Figure 1:**
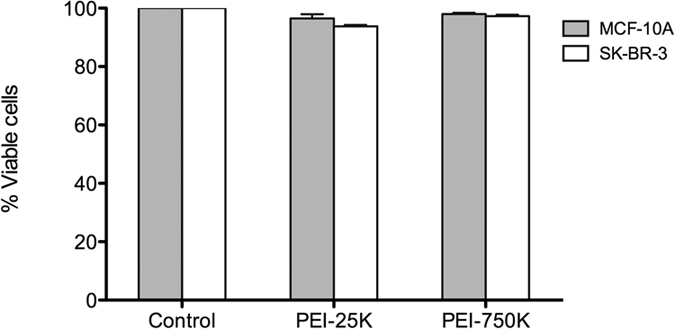


The cytotoxic effect of PEI treatments was evaluated by flow cytometry 24 h after incubation of cells with PEI-25 K or PEI-750 K. Figure 1 shows that the percentage of viable cells, normalized to the control, was higher than 90% in all cases. Moreover, no significant differences (*P *> 0.05) were found between PEI treatments and control cells, indicating that 4 h incubation with either PEI-25 K or PEI-750 K 0.05 mM did not cause cytotoxicity in either SKBR-3 or MCF-10A cells.

### Characterization of microparticles

**Figure 2:**
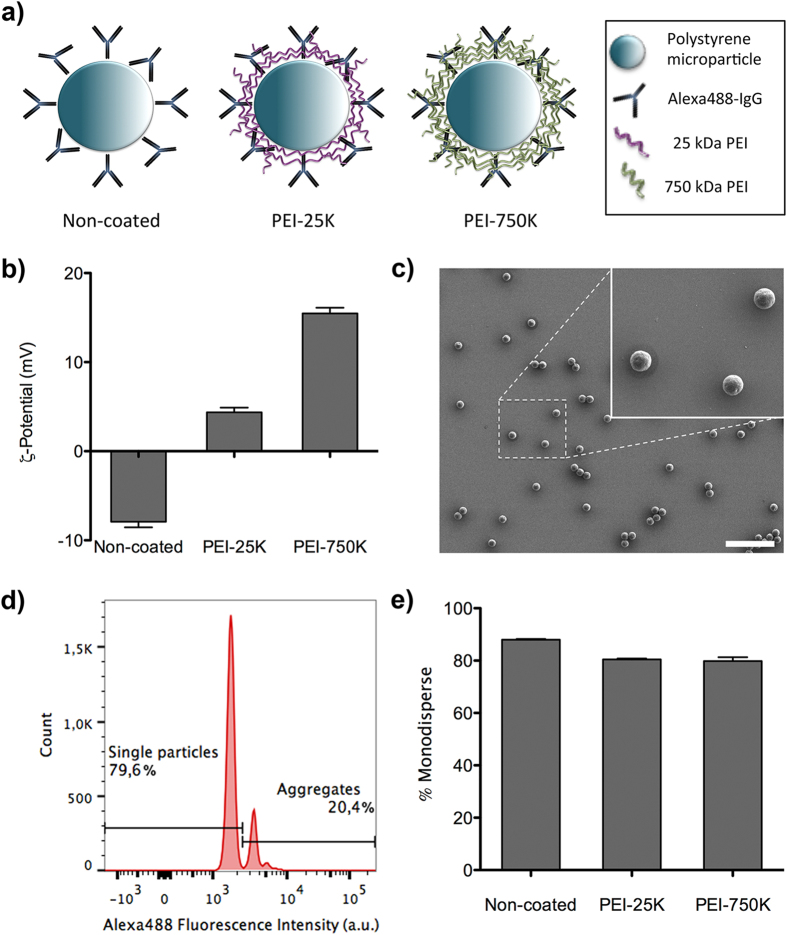
Characterization of Alexa488-IgG functionalized microparticles. **a**) Scheme of Alexa488-IgG microparticles (non-coated) and coated with different molecular weight PEI (PEI-25 K and PEI-750 K, respectively). **b**) ζ-Potential of non-coated and coated (PEI-25 K and PEI-750 K) Alexa488-IgG microparticles. **c**) Representative SEM image of Alexa488-IgG microparticles, in the example coated with PEI-25 K. Scale bar corresponds to 20 μm. Inset shows a magnification of the selected area. **d**) Representative fluorescence intensity histogram of Alexa488-IgG microparticles (in the example coated with PEI-25 K), obtained by flow cytometry analysis. **e**) Percentage of monodisperse, non-coated, PEI-25 K and PEI-750 K Alexa488-IgG microparticles, calculated by flow cytometry analyzed as shown in **d**).

Polystyrene microparticles were functionalized with Alexa Fluor^®^488 conjugated goat anti-Rabbit IgG (Alexa488-IgG) and then coated with different molecular weight Polyethyleneimine (PEI), 25 kDa or 750 kDa (PEI-25 K or PEI-750 K), as described in Fig. 2a. Surface charge of microparticles was evaluated by measuring their ζ-potential (Fig. 2b). Results showed that Alexa488-IgG microparticles were successfully coated with PEI, as their negatively charged surface changed to positively charged, being significantly higher when PEI-750 K was used. In order to qualitatively assess the dispersion status of microparticles, SEM images of microparticles were recorded (Fig. 2c). Although the majority of particles were in a monodispersed form, few aggregates were also observed. To quantify the amount of monodispersed particles, samples were analyzed using a Flow Cytometer, where the amount of fluorescence intensity allowed discriminating between single and aggregated particles (Fig. 2d). Figure 2e shows that in all cases the percentage of monodispersed microparticles was above 80%, which was considered as an acceptable value for our studies.

### Microparticle internalization efficiency

**Figure 3:**
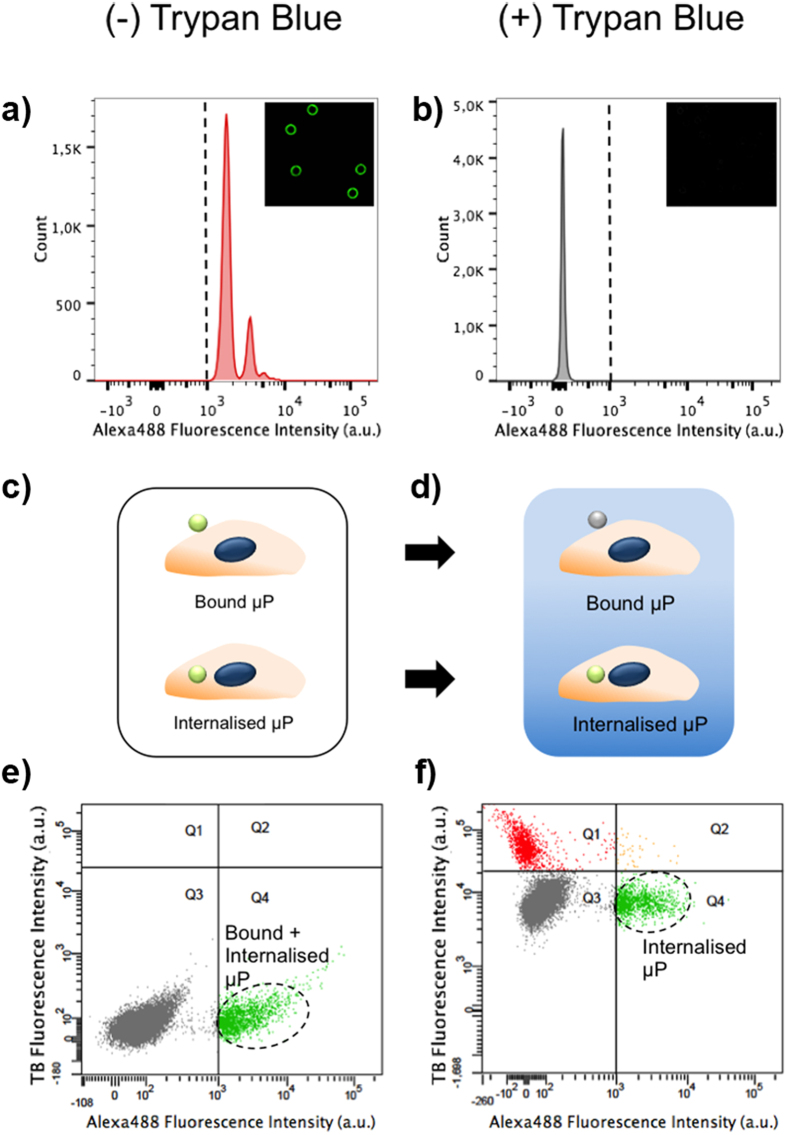
Quenching effect of Trypan Blue (TP) allowing discriminating internalized microparticles from those attached to the cell membrane. **a**,**b**) Fluorescence intensity of Alexa488-IgG microparticles before (**a**) and after (**b**) TB addition, analyzed by flow cytometry. Fluorescence boundary was established with Alexa488-IgG microparticles fluorescence emission (>103 a.u.). Inset shows an image of the same Alexa488-IgG microparticles before and after TB addition. **c,d**) Scheme of extracellular quenching effect by TB. After TB addition (**d**), only Alexa488-IgG microparticles attached to the cell membrane can be quenched, TB cannot quench internalized ones. **e,f**) Representative dot-plot of cells incubated with Alexa488-IgG microparticles, analyzed by flow cytometry after 4 h of incubation before (**e**) and after (**f**) TB addition. In the absence of TB, two populations of cells were observed: cells with Alexa488-IgG microparticles (Q4, green), either attached to the plasma membrane or internalized, and cells without microparticles (Q3, grey). TB can enter only in cells with damaged membrane (dead cells), thus the addition of TB induced the visualization of a new population of cells: those emitting in the red cannel (Q1). Therefore, Q3 and Q4 constitute the population of live cells, being Q3 cells without microparticles or cells with microparticles attached to the membrane that have been quenched, whereas Q4 shows the population of cells that have internalized the microparticles.

Internalization of microparticles by both MCF-10A and SKBR-3 cells was measured by flow cytometry. In order to distinguish cells with membrane-attached microparticles from cells that had completely internalized them, trypan blue (TB) quenching of extracellular microparticles was performed (optimization of TB concentration to completely quench microparticle fluorescence is summarized in supplementary data, figures S1 and S2). Figure 3 shows how the TB quenching effect was used for distinguishing microparticles internalized from those attached to the cell membrane. Fluorescence of Alexa488-IgG-microparticles was analyzed before and after the addition of 2 mg/ml TB (Fig. 3a). We observed that fluorescence was completely eliminated after TB addition.

Figure 3b shows a schematic representation of the extracellular quenching effect by TB. As TB is not capable of penetrating live cells, the quenching effect only occurs in the extracellular space. Thus, the fluorescence of microparticles bound to the membrane can be quenched by TB, whereas the internalized ones remain intact. Figure 3c shows a typical flow cytometry analysis of cells incubated with microparticles (in this case MCF-10A cells incubated with PEI-25 K coated microparticles). Two different populations of cells were observed before TB addition: the population of cells without microparticles (no fluorescence emission) and the population of cells with microparticles, either internalized or bound to the cell membrane (green fluorescence emission). After the addition of TB, the fluorescence of non-internalized microparticles was quenched, allowing us to discriminate between microparticles internalized (green fluorescence) and microparticles adhered to the cell membrane (no fluorescence emission). Moreover, the addition of TB to the cell suspension permitted to determine cell viability, since TB can only enter in cells with damaged plasma membranes. Thus, a third population was observed after the TB addition, corresponding to dead cells which emitted red fluorescence due to the presence of TB in their cytoplasm. In this study, we used the Q4 population, i.e. live cells with internalized microparticles. in order to compare the effect of the different surface modifications of microparticles on their uptake efficiency by SKBR-3 and MCF-10A cells.

**Figure 4:**
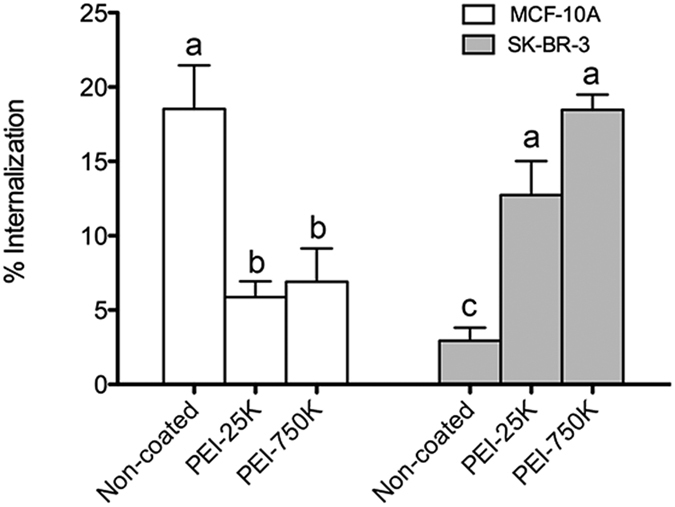
Internalization efficiency of non-coated and PEI-coated Alexa488-IgG microparticles by MCF-10A and SKBR-3 cell lines, analyzed by flow cytometry. Results are representative of three independent experiments and data is shown as the mean ± SEM. Different superscripts **(a–c**) denote groups of significance (P < 0.05).

Figure 4 shows the percentage of live cells with internalized microparticles for both cell lines. We observed that MCF-10A cells internalized non-coated microparticles (Alexa488-IgG-microparticles) with a 3-fold higher efficiency than SKBR-3 cells. By contrast, PEI coating of microparticles resulted in an opposed effect. When PEI coated microparticles (PEI-25 K and PEI-750 K) were used, a significant reduction in microparticle internalization was observed in MCF-10A cells, whereas the opposite effect was observed in SKBR-3 cells. In addition, for both cell lines, although a higher percentage of internalization was observed in the case of PEI-750 K, no significant differences were found when compared to PEI-25 K. All experiments were conducted by incubating the microparticles in serum-free medium.

### Effect of serum and endocytic inhibitors on microparticle internalization

**Figure 5:**
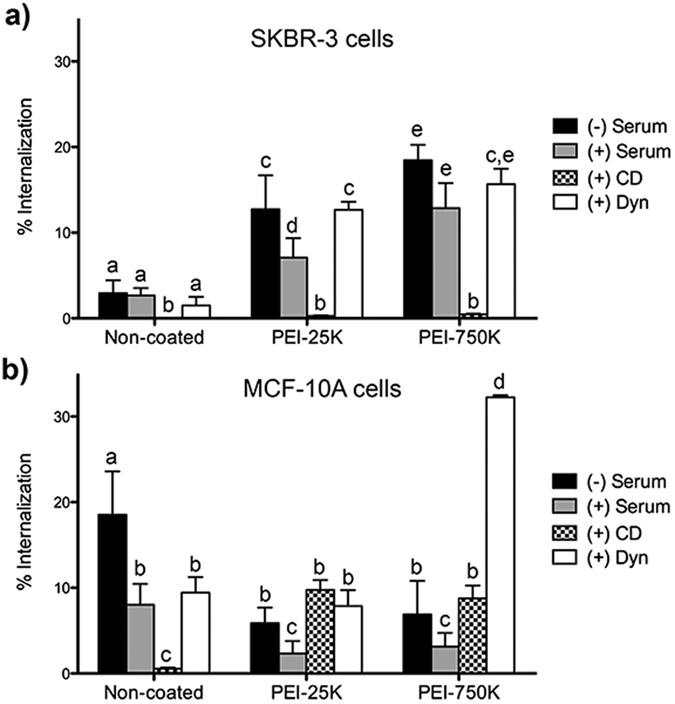
Internalization of Alexa488-IgG functionalized microparticles either coated or not with PEI (25K or 750K) by SKBR-3 (**a**) and MCF-10A (**b**) cells, when incubated with serum, Cytochalasin D (CD) or Dynasore (Dyn). Results are shown as the mean ± SEM. Different superscripts (**a–e**) denote groups of significance (P < 0.05).

The effect of serum and endocytic inhibitors on microparticle internalization was evaluated by flow cytometry, as previously described. Figure 5a,b show the percentages of internalization in SKBR-3 and MCF-10A cells, respectively. For both cell lines, the presence of serum during incubation with microparticles significantly reduced their internalization, except in the case of SKBR-3 in presence of non-coated microparticles and MCF-10A incubated with PEI-750 K coated microparticles, where internalization was not affected.

Regarding endocytosis inhibition, we observed a cell line dependent effect of macropynocitosis inhibition by cytochalasin D (CD). Whereas in SKBR-3 cells the internalization of all types of microparticles was completely inhibited by CD, in MCF-10A cells the CD effect was only detected in the case of non-coated microparticles.

The addition of Dynasore (Dyn) also induced a differential cell response regarding internalization of microparticles. In SKBR-3 cells, Dyn did not affect the internalization of any type of microparticles. By contrast, the incubation with Dyn significantly reduced the uptake of non-coated microparticles by MCF-10A cells. This reduction was not observed in either PEI-25 K or PEI 750 K.

The cytotoxicity of all incubation conditions was determined by the use of TB in flow cytometry analyses. Results showed that none of the incubation conditions altered cell survival when compared to control cells (figure S3).

### Intracellular location of microparticles

**Figure 6:**
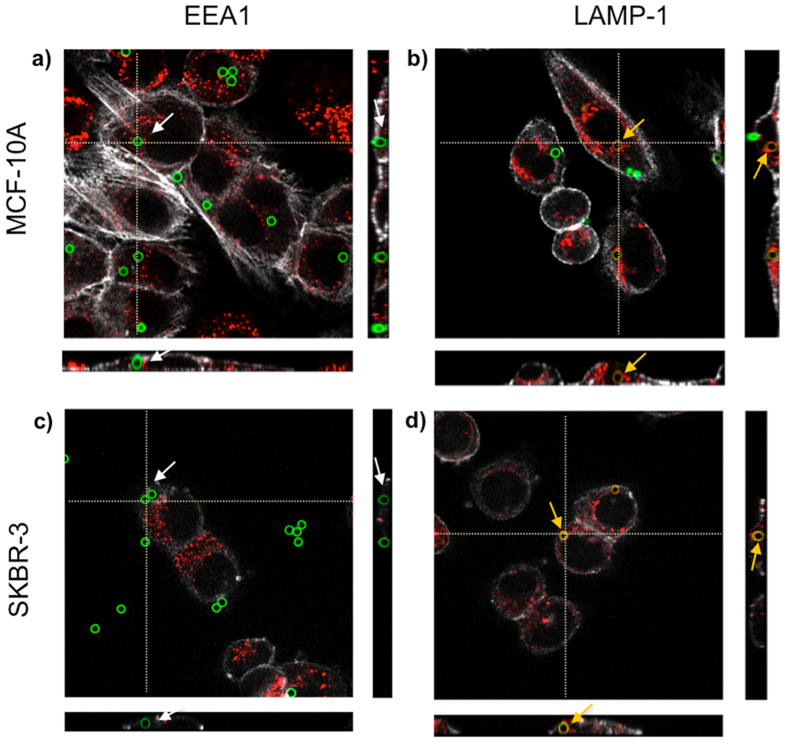
Intracellular location of microparticles, analyzed by Confocal Laser Scanning Microscope (CLSM). Orthogonal projections of z-stacks of MCF10A (**a,b**) or SKBR-3 (**c,d**) cells incubated with the endosome marker EEA1 (red) (**a,c**) or the lysosome marker LAMP-1(red) (**b,d**). Microparticles could be only observed in lysosomes (orange arrows), but not in endosomes (white arrows). In all cases, cell cortex was stained using AlexaFluor®594-conjugated Phalloidin.

In order to determine the intracellular fate of non-coated, PEI-25 K and PEI-750 K microparticles in SKBR-3 and MCF-10A cells, immunolocalization of EEA-1 (endosomal compartment) and LAMP-1 (lysosomal compartment) proteins was carried out. Confocal Laser Scanning Microscope (CSLM) analyses showed that at 4 h the majority of microparticles positively colocalized with LAMP-1 marker, indicating that microparticles were located inside the lysosomal compartment (Fig. 6). These results were found for both cell lines and for all microparticle treatments. Moreover, 24 h later, the majority of microparticles (>80%) remained in the lysosomal compartment.

## Discussion

Although the use of micro- and nanosystems for drug delivery has shown a strong potential for biomedical applications, a deeper understanding of their interaction with cells is required for more efficient design of drug delivery systems. Moreover, whereas a wide range of studies have focused on the study of nanoparticles behavior in contact with cells, less is known about larger particles.

In the present study we compared the response of normal and tumoral breast epithelial cell lines to 3 μm microparticles with different surface properties. First, polystyrene microparticles were covalently conjugated to a fluorescently labelled secondary antibody. Then, the cationic polymer PEI at two different molecular weights was used to coat microparticles in order to 1) further modify their surface properties and, 2) evaluate how this could impact on their interaction with cells. Although PEI has been successfully used for gene delivery and/or functionalization of micro- and nanoparticles^36^,^37^,^38^,^39^, it has been proved to yield cytotoxic effects, depending on its form (linear or branched) and molecular weight^40^.

Thus, the first step in this work was to assess the cytotoxicity of free PEI-25 k and PEI-750K at a 0.05 mM concentration. Given that PEI at that concentration did not trigger cytotoxic effects in either MCF-10A or SKBR-3 cells, microparticles were coated with PEI-25 k and PEI-750 K (0.05 mM). PEI coating clearly changed the surface of microparticles from negatively to positively charged. This change was significantly higher for PEI-750 K than for PEI-25 K, almost certainly due to the former having a higher molecular weight and a higher number of protonable amine groups than the latter.

To evaluate the surface charge effect of the PEI coating of microparticles on cellular uptake, flow cytometry was used to determine the internalization efficiency. Results showed that microparticle internalization was dependent on surface charge in both cell lines, as it has been previously described by other authors^17^,^20^,^41^. However, although it has been widely reported that positively charged microparticles are preferentially uptaken by cells, we observed that MCF-10A cells were more able to internalize negatively charged non-coated microparticles than their PEI-coated counterparts. In contrast, in SKBR-3 cells the uptake increased with positively charged microparticles (PEI-coated), being nearly depreciable for non-coated ones (negatively charged). In fact, it has been reported that MCF-10A cells can internalize both positively^18^ and negatively^26^ charged nanoparticles and the same seems to apply to microparticles. Taken together, our results indicate that surface charge clearly determines microparticle uptake by cells. However, the uptake can be either enhanced or inhibited, depending on the cell type, which suggests that targeting to certain cell types can benefit from the microparticle surface charge. Thus, to identify the behavior of a specific cell line in front a specific particle is very important when designing new particles for drug delivery. Our results indicate that two human cell lines, both coming from the mammary epithelia showed completely different uptake capability depending on their tumorigenic or non-tumorigenic nature.

The presence of serum in the culture medium is also critical for microparticle internalization, as a protein corona can form around micro/nano particles, modifying their surface^42^. In this regard, it is shown that the presence of serum had an inhibitory internalization effect on both cell lines and for all types of microparticles, except for internalization of non-coated microparticles by SKBR-3 cells and PEI-750 K coated microparticles by MCF-10A This finding highlights the impact of cell type on microparticle-cell interactions, and is in agreement with Yan *et al*. (2013), who observed a differential impact of protein corona in THP-1 monocytic cells and macrophages^43^.

In order to further investigate whether the mechanism of internalization was also dependent on the cell line and surface properties of microparticles, two endocytosis inhibitors, CD and Dyn, were used. CD has been reported to mainly inhibit macropinocytosis, whereas Dyn causes inhibition of dynamin-dependent endocytosis^44^, i.e. clathrin- and caveolin-mediated endocytosis. In the case of the SKBR-3 line, microparticle internalization was inhibited almost completely by CD but not by Dyn, both for non-coated and PEI-coated microparticles, indicating that in these cells microparticle internalization occurs by macropinocytosis rather than by dynamin-dependent mechanisms. It has been reported that SKBR-3 cells internalize their overexpressed ErbB2 receptors in a clathrin- and caveolin- independent endocytic pathway^45^. What is more, SKBR-3 cells lack caveolae, and ErbB2 internalization is sensitive to the depletion of cholesterol, which is necessary for most of the clathrin-independent pathways^46^. In addition, an association between macropinocytosis and tumor progression/metastasis has been reported^47^. Altogether, these studies could explain why SKBR-3 cells use the macropinocytosis pathway to internalize all types of microparticles, regardless of their surface properties.

**Figure 7:**
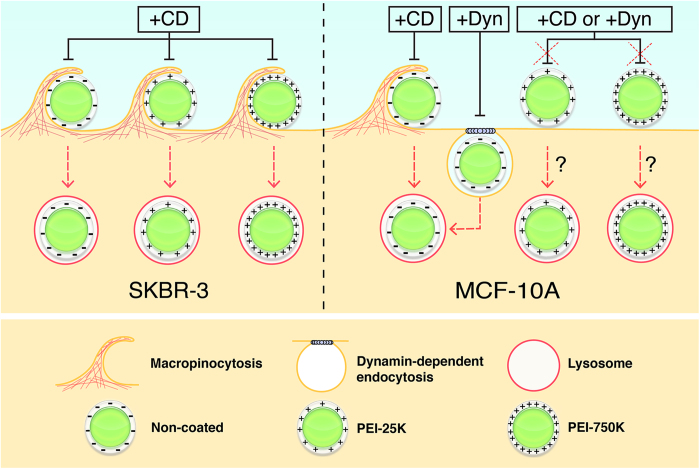
Scheme of the differential mechanisms of microparticle internalisation by SKBR-3 and MCF-10A cells. SKBR-3 cells internalized all types of microparticles by macropinocytosis, as their internalization was only inhibited by Cytochalasin D (CD). By contrast, MCF-10A cells showed different mechanisms of internalization, depending on the microparticle type. Non-coated Alexa488-IgG microparticles could be taken up by both macropinocytosis and dynamin-dependent endocitosis, as their internalization was inhibited by both CD and Dynasore (Dyn). By contrast, the uptake of PEI-25 K and PEI-750 K coated microparticles was not affected by either CD or Dyn, indicating that they are taken up by alternative mechanisms.

By contrast, in MCF-10A cells, both CD and Dyn inhibited negatively charged microparticle uptake, suggesting that both macropinocitosis and dynamin-dependent endocytosis were involved. On the other hand, internalization of PEI-coated microparticles by MCF-10A cells was not affected by either CD or Dyn, suggesting that positively charged microparticles are internalized through alternative mechanisms in these cells. In addition, a higher percentage of PEI-750 K microparticles were internalized in the presence of Dyn than in its absence. This fact could be attributed to a compensatory effect, as it has been previously described that clathrin and caveolae dependent endocytosis inhibition can cause stimulation of macropinocytosis of positively charged nanoparticles^22^. However, further studies are needed to better understand this particular result. Taken together, all these findings suggest that both the surface charge and the cell type have a strong impact not only on the uptake efficiency, but also on the mechanism of microparticle internalization (Fig. 7).

Finally, we assessed the intracellular location of microparticles in order to evaluate whether surface charge or cell type could affect the intracellular fate of the microparticles. Results showed that in all cases microparticles were located inside the lysosomal compartment, regardless of their surface properties and cell type. This suggests that despite the fact that PEI proton-sponge effect allows the delivery of DNA and small nanoparticles to the cytosol^48^, it is not capable of releasing large microparticles into the cytosol. In this regard, future research should be focused on alternative approaches for the efficient delivery of desired microparticles or molecules into the cytosol.

In summary, we have demonstrated that normal and tumor cells use different endocytic pathways when internalizing different types of modified microparticles. The tumor cell line SKBR-3 internalizes, mainly, positively charged microparticles, using the macropinocytosis pathway. When this pathway is inhibited, internalization is interrupted. By contrast, the non-tumor cell line MCF-10A uses different pathways to predominantly internalize negative microparticles. It seems that there is not a predominant uptake mechanism, and that compensatory internalization pathways are used when one of the pathways is inhibited. Regardless of the cell line and microparticle surface, the fate of the microparticles is always the lysosome compartment, even in presence of PEI-750 K. Thus new strategies, as pH sensitive cargo-link or esterase sensitive cargo-link, among others, must be envisaged to allow the liberation of the cargo to the cytoplasm. Consequently, the design of microcarriers against specific tissues must be done taken into account i) the surface of the microparticle and ii) the characteristics of the target cell, i.e., the uptake pathway and the final fate.

## Methods

### Functionalization of microparticles

Carboxylated microparticles (Polybead® Carboxylate Microspheres 3 μm; Polysciences, Inc, Warrington, PA) were functionalized with an Alexa Fluor® 488 conjugated goat anti-Rabbit IgG H&L antibody (Life Technologies, Carlsbad, CA) using the PolyLink Protein Coupling kit (Polysciences, Inc.), following the manufacturer’s instructions (Alexa488-IgG-microparticles, from now on).

### PEI coating and characterization of microparticles

To coat Alexa488-IgG microparticles with PEI, 5 μL of PEI-25 K or PEI-750 K (10 mM) were diluted in 135 μl of NaCl. In parallel, 3 μL of microparticle solution (3 × 10^6^ microparticles) was diluted in 137 μL of NaCl. Then, the PEI solution was added to the microparticle solution, mixed slowly and left at room temperature (RT) for 40 min in order to form the PEI-Alexa488-IgG-microparticles complexes. After incubation, 720 μl of serum-free culture medium was added to the solution to obtain a final 0.05 mM concentration of PEI. Non-coated functionalized (Alexa488-IgG-microparticles) and non-functionalized (-COOH microparticles) microparticles, were treated in the same way, but without PEI addition. ζ-Potential of microparticles was measured using a Zetasizer Nano ZS (Malvern Instruments, Malvern, UK). Images of microparticles were recorded using a Scanning Electron Microscope (Zeiss Merlin, Jena, Germany). Fluorescence intensity of microparticles was analyzed using a Becton Dickinson FACSCanto II flow cytometer (BD Biosciences, Franklin Lakes, NJ) equipped with BD Biosciences FACSDiva™ software, in order to quantitatively determine the dispersion state of microparticles.

### Cell culture

Experiments were conducted with two different human mammary epithelial cell lines, a non-tumorigenic (MCF-10A) and a metastatic one (SKBR-3). The MCF-10A cell line (ATCC) was cultured in DMEM/F12 (Gibco, Paisley, United Kingdom) supplemented with 5% horse serum (Gibco), 20 ng/ml epidermal growth factor (Gibco), 0.5 mg/ml hydrocortisone (Sigma-Aldrich, St Louis, MO, USA), 100 ng/ml cholera toxin (Sigma) and 10 μg/ml insulin (Gibco). The SKBR-3 adenocarcinoma cell line (ATCC) was cultured in McCoy’s 5A modified medium (Gibco) supplemented with 10% fetal bovine serum (Gibco). Both cell lines were maintained at 37 °C and 5% CO_2_ (standard conditions).

### Polyethylenimine cytotoxicity analysis

PEI with a molecular weight of 25 kDa (PEI-25K) and 750 kDa (PEI-750K) were purchased from Sigma and a 10 mM stock solution was prepared as previously described^48^.

MCF-10A and SKBR-3 cells were seeded in 35 mm Petri dishes (Nalge Nunc Int, Roskilde, Denmark) at a density of 1.5 × 10^5^ cells/dish. After 48 h, culture medium was replaced with serum-free culture medium containing 0.05 mM of PEI-25 K or PEI-750 K and cells were maintained in standard conditions. Control cells were treated in the same way but in absence of PEI. After 4 h of incubation, the medium was replaced by fresh supplemented culture medium and the cells were incubated again for 24 h. Finally, cells were harvested by trypsinization and cytotoxicity was evaluated using the “LIVE/DEAD® Viability/Cytotoxicity Kit for mammalian cells” (Life Technologies, Carlsbad, CA), following the manufacturer’s instructions. Cells were analyzed under a Becton Dickinson FACSCanto II flow cytometer (BD Biosciences, Franklin Lakes, NJ) equipped with BD Biosciences FACSDiva™ software. For each treatment, four independent experiments were performed, where 20.000 cells were analyzed.

### Microparticle internalization

MCF-10A and SKBR-3 cells were seeded in 35 mm petri dishes at a density of 1.5 × 10^5^ cells/dish. After 48 h, the medium was removed and cells were incubated with Alexa488-IgG-microparticles either non-coated, or coated with PEI-25 K or PEI-750 K at a 5:1 ratio (microparticle:cell), in serum-free medium for 4 h in standard conditions. Microparticle internalization was evaluated at 4 h, when cells were harvested by trypsinization and analyzed under a flow cytometer. In order to distinguish internalized microparticles from those attached to the cell membrane, 2 mg/ml Trypan Blue (TB, Sigma) was used to quench the extracellular fluorescence^13^,^21^. Cells were analyzed under a Becton Dickinson FACSCanto II flow cytometer both prior and after TB addition. This method allowed obtaining integrated information about cell viability and microparticle internalization efficiency (% of cells with internalized microparticles). For each PEI treatment and time of incubation, three independent experiments were performed analyzing 20.000 cells each.

### Endocytosis inhibition

To elucidate the mechanism of microparticles internalization, two endocytosis inhibitors were used: Cytochalasin D (CD; macropinocitosis inhibition) and Dynasore (Dyn; Dynamin-dependent endocytosis inhibition). MCF-10A and SKBR-3 cells were seeded in 35 mm petri dishes at a density of 1.5 × 10^5^ cells/dish. At 48 h, prior to the addition of microparticles, cells were pre-incubated for 1 h with 10 μg/ml CD (Sigma-Aldrich), or 80 μg/ml Dyn (Sigma-Aldrich) in serum-free medium. After that, pre-incubation medium was removed and cells were exposed to non-coated Alexa488-IgG-microparticles, PEI-25 K or PEI-750 K coated Alexa488-IgG-microparticles at a 5:1 ratio (microparticle:cell) in presence of the endocytosis inhibitors (10 μg/ml CD, or 80 μg/ml Dyn) for 4 h. Finally, cells were recovered by trypsinization and analyzed under a Becton Dickinson FACSCanto II flow cytometer.

In addition, the effect of serum on microparticle internalization was tested by incubating the microparticles (non-coated and PEI-coated) in culture medium containing serum. After 4 h of incubation, the cells were trypsinized and microparticle internalization was evaluated by flow cytometry.

### Immunofluorescence detection of endosomal and lysosomal compartments

Cells were seeded on coverslips in 4-well culture dishes at a density of 30,000 cells/well. After 48 h, they were exposed to functionalized microparticles, either non-coated or coated with PEI-25 K or PEI-750 K, at a 5:1 ratio (microparticle:cell) and incubated for 4 h. Then, cells were washed twice with PBS, fixed with 4% paraformaldehyde/PBS (Sigma-Aldrich), permeabilized with 0.1% Triton X-100 (Sigma-Aldrich) in PBS, and blocked with 5% PBS-BSA (Sigma-Aldrich) for 40 min. Cells were incubated for 1 h at 37 °C with either mouse anti-EEA-1 monoclonal antibody (BD Biosciences, Franklin Lakes, NJ) or mouse anti-LAMP-1 polyclonal antibody (BD Biosciences), in order to label endosomal or lysosomal compartments, respectively. After that, cells were washed thrice with PBS and incubated at RT for 1 h with Cy5-conjugated chicken anti-mouse IgG antibody (Life technologies). Finally, cells were washed twice with PBS and incubated with Alexa 594-Conjugated Phalloidin (5 ug/ml, Life Technologies) and Hoechst 33258 (0.5 ug/ml, Life Technologies) to stain actin and DNA, respectively, prior to their analysis under a Confocal Laser Scanning Microscope (CLSM, Olympus XT7). For each labelling, treatment, and time of incubation with microparticles, at least 20 cells were analyzed.

### Statistical analyses

Statistical analyses were conducted using a statistical package (IBM SPSS® for Windows, version 20.0; SPSS Inc., Chicago, IL, USA). Data were first tested for normality (Shapiro-Wilk test) and homogeneity of variances (Levene test). When necessary, data (x) in percentages were arcsine-transformed (arcsine √x) to accomplish the parametric assumptions (i.e. normal distribution and variance homogeneity).

A series of separate general linear models (analysis of variance, ANOVA) were run to test the effects of coated and non-coated microparticles and/or PEI concentrations on **ζ**-potential, cell viability and percentage of internalization. Briefly, the effects of PEI treatments on SKBR-3 and MCF-10A viability and internalization were determined through a two-way ANOVA (factors: PEI treatment and cell line, SKBR-3 and MCF-10A; variables: % cell viability or % internalization), followed by a post-hoc Sidak test, whereas **ζ**-Potential of carboxylated microparticles non functionalized (-COOH), Alexa 488-IgG functionalized microparticles (488IgG), Alexa488-IgG-microparticles coated with PEI25K (PEI25K) or PEI750K (PEI750K) was compared by a one-way ANOVA followed by a post-hoc Sidak test. Finally, the effects on non-coated, PEI-25 kDa-, PEI750kDa-coated microparticles intake of incubation with or without serum, or with endocytosis inhibitors (CD or Dyn) were tested in MCF-10A and SKBR-3 cells through a three-way ANOVA (factors: medium composition, PEI treatment, and cell line; variable: % internalization), again followed by a post-hoc Sidak test.

In all cases, the level of significance was set at P < 0.05. Data are presented as mean ± standard error of the mean (SEM).

## Additional Information

**How to cite this article**: Patiño, T. *et al*. Surface modification of microparticles causes differential uptake responses in normal and tumoral human breast epithelial cells. *Sci. Rep*. **5**, 11371; doi: 10.1038/srep11371 (2015).

## Supplementary Material

Supplementary Information

## References

[b1] Carregal-Romero S. . Multiplexed sensing and imaging with colloidal nano- and microparticles. Annu. Rev. Anal. Chem. (Palo Alto. Calif). 6, 53–81 (2013). 23451718 10.1146/annurev-anchem-062012-092621

[b2] Cederquist K. B., Dean S. L. & Keating C. D. Encoded anisotropic particles for multiplexed bioanalysis. Wiley Interdiscip. Rev. Nanomed. Nanobiotechnol. 2, 578–600 (2010). 20890960 10.1002/wnan.96

[b3] Sen T. & Bruce I. J. Surface engineering of nanoparticles in suspension for particle based bio-sensing. Sci. Rep. 2, 564 (2012). 22872809 10.1038/srep00564 PMC3413008

[b4] Anker J. N. . Biosensing with plasmonic nanosensors. Nat. Mater. 7, 442–53 (2008). 18497851 10.1038/nmat2162

[b5] Yu M. K., Park J. & Jon S. Targeting strategies for multifunctional nanoparticles in cancer imaging and therapy. Theranostics 2, 3–44 (2012). 22272217 10.7150/thno.3463 PMC3263514

[b6] Sanna V., Pala N. & Sechi M. Targeted therapy using nanotechnology: focus on cancer. Int. J. Nanomedicine 9, 467–483 (2014). 24531078 10.2147/IJN.S36654 PMC3896284

[b7] Gu F. X. . Targeted nanoparticles for cancer therapy. Nano Today 2, 14–21 (2007).

[b8] Jain N. K., Mishra V. & Mehra N. K. Targeted drug delivery to macrophages. Expert Opin. Drug Deliv. 10, 353–67 (2013). 23289618 10.1517/17425247.2013.751370

[b9] Caldorera-Moore M. & Peppas N. a. Micro- and nanotechnologies for intelligent and responsive biomaterial-based medical systems. Adv. Drug Deliv. Rev. 61, 1391–401 (2009). 19758574 10.1016/j.addr.2009.09.002 PMC2788786

[b10] Cai Y., Chen Y., Hong X., Liu Z. & Yuan W. Porous microsphere and its applications. Int. J. Nanomedicine 8, 1111–20 (2013). 23515359 10.2147/IJN.S41271 PMC3600995

[b11] Mura S., Nicolas J. & Couvreur P. Stimuli-responsive nanocarriers for drug delivery. Nat. Mater. 12, 991–1003 (2013). 24150417 10.1038/nmat3776

[b12] Zauner W., Farrow N. a. & Haines, *In vitro* uptake of polystyrene microspheres: effect of particle size, cell line and cell density. J. Control. Release 71, 39–51 (2001). 11245907 10.1016/s0168-3659(00)00358-8

[b13] Rejman J., Oberle V., Zuhorn I. S. & Hoekstra D. Size-dependent internalization of particles via the pathways of clathrin- and caveolae-mediated endocytosis. Biochem. J. 377, 159–69 (2004). 14505488 10.1042/BJ20031253 PMC1223843

[b14] Pacheco P., White D. & Sulchek T. Effects of microparticle size and Fc density on macrophage phagocytosis. PLoS One 8, e60989 (2013). 23630577 10.1371/journal.pone.0060989 PMC3632606

[b15] Shang L., Nienhaus K. & Nienhaus G. U. Engineered nanoparticles interacting with cells: size matters. J. Nanobiotechnology 12, 5 (2014). 24491160 10.1186/1477-3155-12-5 PMC3922601

[b16] Barua S. . Particle shape enhances specificity of antibody-displaying nanoparticles. Proc. Natl. Acad. Sci. USA 110, 3270–5 (2013). 23401509 10.1073/pnas.1216893110 PMC3587278

[b17] Gratton S. E. a. *et al*. The effect of particle design on cellular internalization pathways. Proc. Natl. Acad. Sci. USA 105, 11613–8 (2008). 18697944 10.1073/pnas.0801763105 PMC2575324

[b18] Saha K. . Surface functionality of nanoparticles determines cellular uptake mechanisms in mammalian cells. Small 9, 300–5 (2013). 22972519 10.1002/smll.201201129 PMC4070423

[b19] Dausend J. . Uptake mechanism of oppositely charged fluorescent nanoparticles in HeLa cells. Macromol. Biosci. 8, 1135–43 (2008). 18698581 10.1002/mabi.200800123

[b20] Fröhlich E. The role of surface charge in cellular uptake and cytotoxicity of medical nanoparticles. Int. J. Nanomedicine 7, 5577–91 (2012). 23144561 10.2147/IJN.S36111 PMC3493258

[b21] Thiele L., Merkle H. P. & Walter E. Phagocytosis and phagosomal fate of surface-modified microparticles in dendritic cells and macrophages. Pharm. Res. 20, 221–8 (2003). 12636160 10.1023/a:1022271020390

[b22] Harush-Frenkel O., Debotton N., Benita S. & Altschuler Y. Targeting of nanoparticles to the clathrin-mediated endocytic pathway. Biochem. Biophys. Res. Commun. 353, 26–32 (2007). 17184736 10.1016/j.bbrc.2006.11.135

[b23] Patiño T., Nogués C., Ibáñez E. & Barrios L. Enhancing microparticle internalization by nonphagocytic cells through the use of noncovalently conjugated polyethyleneimine. Int. J. Nanomedicine 7, 5671–5682 (2012). 23152683 10.2147/IJN.S34635 PMC3496409

[b24] Boussif O. . A versatile vector for gene and oligonucleotide transfer into cells in culture and *in vivo*: polyethylenimine. Proc. Natl. Acad. Sci 92, 7297–7301 (1995). 7638184 10.1073/pnas.92.16.7297 PMC41326

[b25] Yu J.-H., Quan J.-S., Huang J., Nah J.-W. & Cho C.-S. Degradable poly(amino ester) based on poly(ethylene glycol) dimethacrylate and polyethylenimine as a gene carrier: molecular weight of PEI affects transfection efficiency. J. Mater. Sci. Mater. Med. 20, 2501–10 (2009). 19597971 10.1007/s10856-009-3816-z

[b26] Xiao Y. . Dynamics and mechanisms of quantum dot nanoparticle cellular uptake. J. Nanobiotechnology 8, 13 (2010). 20550705 10.1186/1477-3155-8-13 PMC2898766

[b27] Oliveira S., Heukers R., Sornkom J., Kok R. J. & van Bergen En Henegouwen, P. M. P. Targeting tumors with nanobodies for cancer imaging and therapy. J. Control. Release 172, 607–617 (2013). 24035975 10.1016/j.jconrel.2013.08.298

[b28] Patino T. . Multifunctional gold nanorods for selective plasmonic photothermal therapy in pancreatic cancer cells using ultra-short pulse near-infrared laser irradiation. Nanoscale 7, 5328–5337 (2015). 25721177 10.1039/c5nr00114e

[b29] Conniot J. . Cancer immunotherapy: nanodelivery approaches for immune cell targeting and tracking. Front. Chem. 2, 105 (2014). 25505783 10.3389/fchem.2014.00105 PMC4244808

[b30] Loureiro J. A., Gomes B., Coelho M. A. N., do Carmo Pereira M. & Rocha S. Targeting nanoparticles across the blood-brain barrier with monoclonal antibodies. Nanomedicine (Lond). 9, 709–722 (2014). 24827845 10.2217/nnm.14.27

[b31] Ferrati S. . Inter-endothelial transport of microvectors using cellular shuttles and tunneling nanotubes. Small 8, 3151–60 (2012). 22930522 10.1002/smll.201200472 PMC12110420

[b32] Palankar R. . Controlled intracellular release of peptides from microcapsules enhances antigen presentation on MHC class I molecules. Small 5, 2168–76 (2009). 19644923 10.1002/smll.200900809

[b33] Sukhorukov G. B. . Multifunctionalized polymer microcapsules: novel tools for biological and pharmacological applications. Small 3, 944–955 (2007). 17487898 10.1002/smll.200600622

[b34] Delcea M. . Multicompartmental Micro- and Nanocapsules: Hierarchy and Applications in Biosciences. Macromol. Biosci. 10, 465–474 (2010). 20166231 10.1002/mabi.200900359

[b35] Salonen J. . Mesoporous silicon microparticles for oral drug delivery: Loading and release of five model drugs. J. Control. Release 108, 362–374 (2005). 16169628 10.1016/j.jconrel.2005.08.017

[b36] Skandrani N. . Lipid nanocapsules functionalized with polyethyleneimine for plasmid DNA and drug co-delivery and cell imaging. Nanoscale 6, 7379–90 (2014). 24871584 10.1039/c4nr01110d

[b37] Kasturi S. P., Sachaphibulkij K. & Roy K. Covalent conjugation of polyethyleneimine on biodegradable microparticles for delivery of plasmid DNA vaccines. Biomaterials 26, 6375–6385 (2005). 15913771 10.1016/j.biomaterials.2005.03.043

[b38] Ateh D. D. . The intracellular uptake of CD95 modified paclitaxel-loaded poly(lactic-co-glycolic acid) microparticles. Biomaterials 32, 8538–47 (2011). 21824652 10.1016/j.biomaterials.2011.07.060

[b39] McBain S. C., Yiu H. H. P., El Haj a. & Dobson J. Polyethyleneimine functionalized iron oxide nanoparticles as agents for DNA delivery and transfection. J. Mater. Chem. 17, 2561 (2007).

[b40] Neu M., Fischer D. & Kissel T. Recent advances in rational gene transfer vector design based on poly(ethylene imine) and its derivatives. J. Gene Med. 7, 992–1009 (2005). 15920783 10.1002/jgm.773

[b41] Qiu Y. . Surface chemistry and aspect ratio mediated cellular uptake of Au nanorods. Biomaterials 31, 7606–19 (2010). 20656344 10.1016/j.biomaterials.2010.06.051

[b42] Wang F. . The biomolecular corona is retained during nanoparticle uptake and protects the cells from the damage induced by cationic nanoparticles until degraded in the lysosomes. Nanomedicine 9, 1159–68 (2013). 23660460 10.1016/j.nano.2013.04.010

[b43] Yan Y. . Differential roles of the protein corona in the cellular uptake of nanoporous polymer particles by monocyte and macrophage cell lines. ACS Nano 7, 10960–70 (2013). 24256422 10.1021/nn404481f

[b44] Macia E. . Dynasore, a cell-permeable inhibitor of dynamin. Dev. Cell 10, 839–50 (2006). 16740485 10.1016/j.devcel.2006.04.002

[b45] Barr D. J., Ostermeyer-Fay A. G., Matundan R. A. & Brown D. A. Clathrin-independent endocytosis of ErbB2 in geldanamycin-treated human breast cancer cells. J. Cell Sci. 121, 3155–3166 (2008). 18765569 10.1242/jcs.020404 PMC2707784

[b46] Vaidyanath A. . Enhanced internalization of ErbB2 in SK-BR-3 cells with multivalent forms of an artificial ligand. J. Cell. Mol. Med. 15, 2525–2538 (2011). 21323863 10.1111/j.1582-4934.2011.01277.x PMC3822962

[b47] Lim J. P. & Gleeson P. A. Macropinocytosis: an endocytic pathway for internalising large gulps. Immunol. Cell Biol. 89, 836–843 (2011). 21423264 10.1038/icb.2011.20

[b48] Bousiff O. . A versatile vector for gene and oligonucleotide transfer into cells in culture and *in vivo*: Polyethylenimine. 92, 7297–7301 (1995). 10.1073/pnas.92.16.7297 PMC41326 7638184

