# Supplementary material for: Surface modification of microparticles causes differential uptake responses in normal and tumoral human breast epithelial cells
Source: Sci Rep. 2015 Jun 12;5:11371. doi: 10.1038/srep11371 (PMC5155550; doi:10.1038/srep11371)
Supplement: Supplementary Information [file srep11371-s1.pdf]

Title:

Surface modification of microparticles causes differential uptake responses in normal and tumoral human breast epithelial cells

Authors:

Tania Patiño, Jorge Soriano, Lleonard Barrios, Elena Ibáñez, Carme Nogués*

Affiliation:

Unitat de Biologia Cel·lular, Departament de Biologia Cel·lular, Fisiologia i Immunologia. Facultat de Biociències. Universitat Autònoma de Barcelona, 08139 Bellaterra, Spain.

Correspondence:

*Carme Nogués, PhD.

Unitat de Biologia Cel·lular, Departament de Biologia Cel·lular, Fisiologia i Immunologia. Facultat de Biociències.

Edifici C, Campus de Bellaterra

Universitat Autònoma de Barcelona

08193 Bellaterra, Spain

Phone: +34 93 581 2667 Fax:

E-mail: carme.nogues@uab.cat

Supporting information

Optimization of Trypan Blue concentration

Optimization of Trypan Blue (TB) concentration to completely quench microparticle fluorescence was carried out by the addition of increasing concentrations of TB to Alexa488-IgG functionalized microparticles at a concentration of 106 microparticles/ml. Fluorescence intensity in the presence of TB was analysed under a Becton Dickinson FACSCanto II flow cytometer. In figure S1, the decrease in fluorescence intensity is shown. A concentration of 2 mg/ml of TB was sufficient to completely quench microparticles fluorescence. At this TB concentration, the percentage of microparticles emittiong fluorescence was nearly zero (Figure S2).

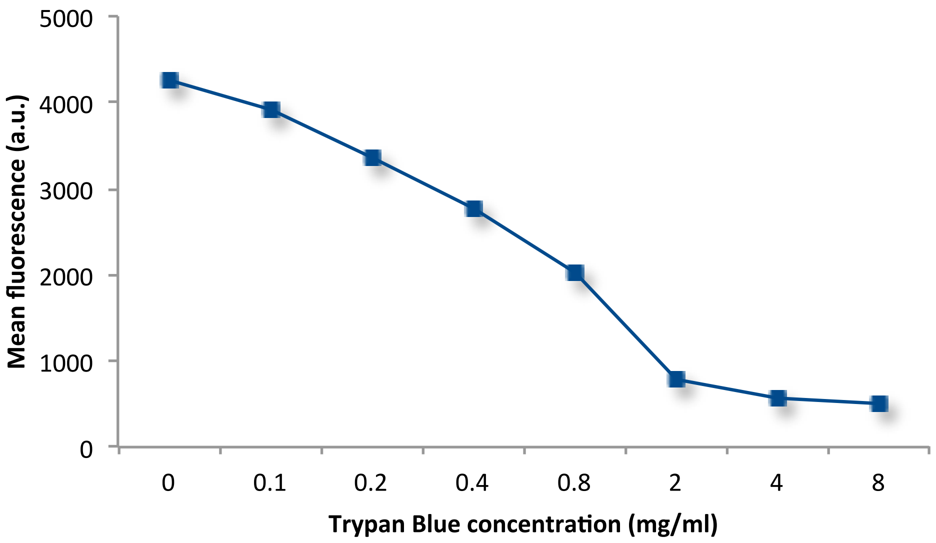

S1. Mean fluorescence of Alexa-488 IgG functionalized microparticles when treated with increasing concentrations of trypan blue, measured by flow cytometry.

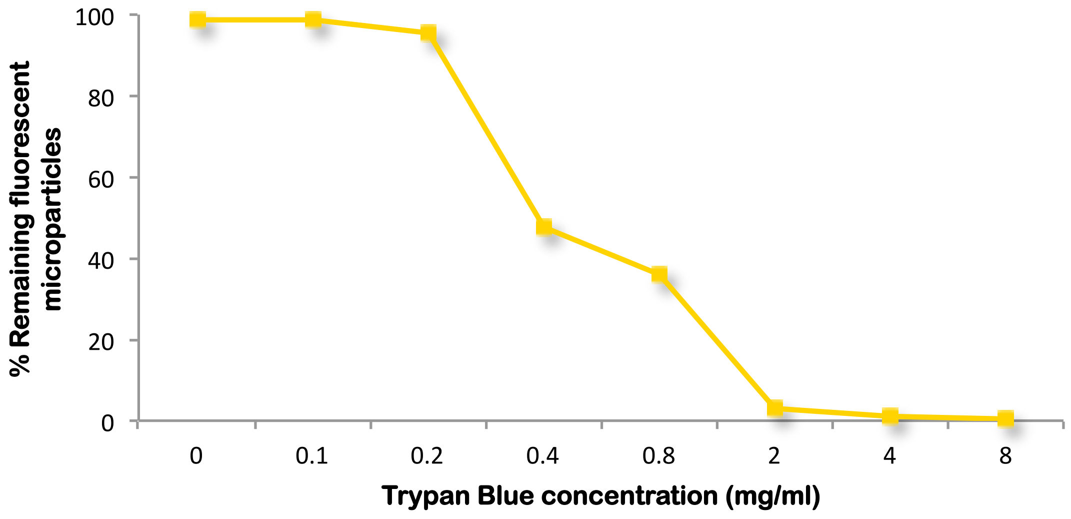

S2. Percentage of microparticles that remained fluorescent after the addition of Trypan Blue at different concentrations, measured by flow cytometry.

Cytotoxicity evaluation of the endocytic inhibitors

In order to find out if the endocytic inhibitors used in the present study, i.e. Cytochalasin D (CD) and Dynasore (Dyn), were cytotoxic or the absence of serum was critical for the viability of the cell lines used in this study (MCF-10A and SKBR-3), cells were seeded in 35 mm petri dishes at a density of 1.5 x 105 cells/dish. At 48 h, prior to the addition of microparticles, cells were pre-incubated for 1 h with 10 µg/ml CD, or 80 µg/ml Dyn in serum-free medium. After that, pre-incubation medium was removed and cells were exposed to non-coated Alexa488-IgG-microparticles, PEI-25K or PEI-750K coated Alexa488-IgG-microparticles in presence of the endocytosis inhibitors (10 µg/ml CD, or 80 µg/ml Dyn) for 4h. Finally, cells were harvested by trypsinization and cytotoxicity was evaluated using the 'LIVE/DEAD® Viability/Cytotoxicity Kit for mammalian cells' (Life Technologies), following the manufacturer's instructions. Cells were analyzed under a Becton Dickinson FACSCanto II flow cytometer (BD Biosciences, Franklin Lakes, NJ) equipped with BD Biosciences FACSDiva™ software. For each treatment, four independent experiments were performed, where 20.000 cells were analyzed.

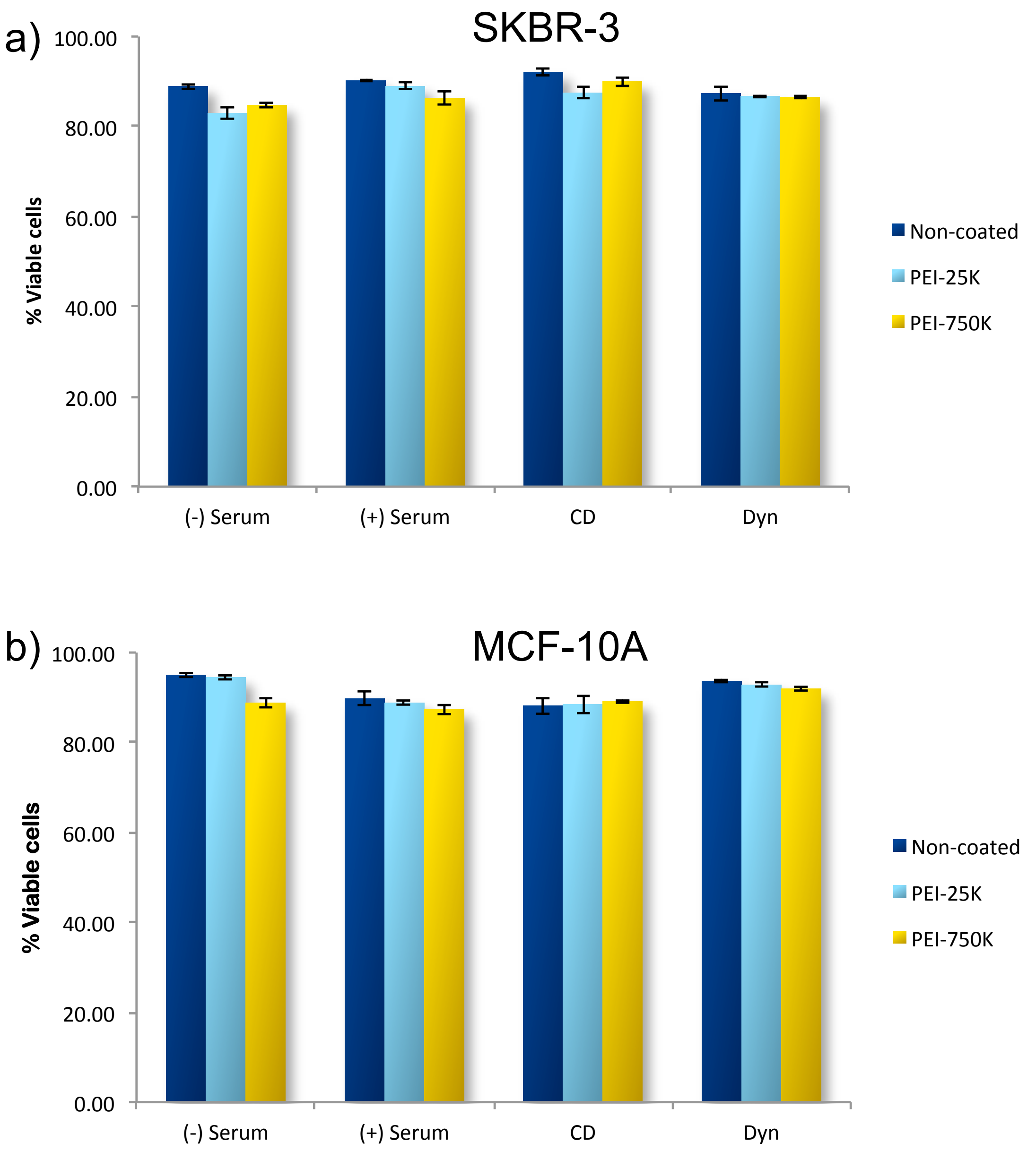

S3. Viability of a) SKBR-3 and b) MCF-10A cells incubated with PEI coated (PEI-25K or PEI-750K) or non-coated Alexa488-IgG-microparticles, in the absence (-serum) or presence (+serum) of serum, and endocityc inhibitors: cytochalasin D (CD) or dynasore (Dyn). Results are shown as the Mean +- SEM. No significant differences were found among groups.

Flow cytometry analyses of internalized microparticles

Internalization of microparticles by both SKBR-3 and MCF-10A cells was analyzed by flow cytometry. First, before trypan blue (TB) addition, populations of cells and free microparticles in solution were distinguished through their respective forward and side scatter distributions (Figure S4-a). Then, microparticle fluorescence intensity was measured (Figure S4-b) and used as a threshold to distinguish between cells with and without microparticles (Figure S4-c and d). Percentages are given with respect to the total number of cells, i.e. other events corresponding to non-cell population were gated out and excluded from the percentage calculation. Once the percentage of cells with or without microparticles was determined, TB quenching was carried out, by adding TB to the samples. Then, all analyses were performed as above described. First, fluorescence of free microparticles population was analyzed, and it was shown that after TB addition, their fluorescence was completely quenched (see Figure 3, main text of the article). Then, cell population was analyzed. TB can only enter cells with damaged membranes (i.e. dead cells), and it cannot penetrate live cell membranes. For this reason, extracellular microparticles fluorescence could be quenched, whereas that of the internalized ones remained intact. In addition, the technique allowed distinguishing live and dead cells, the later emitting red fluorescence.

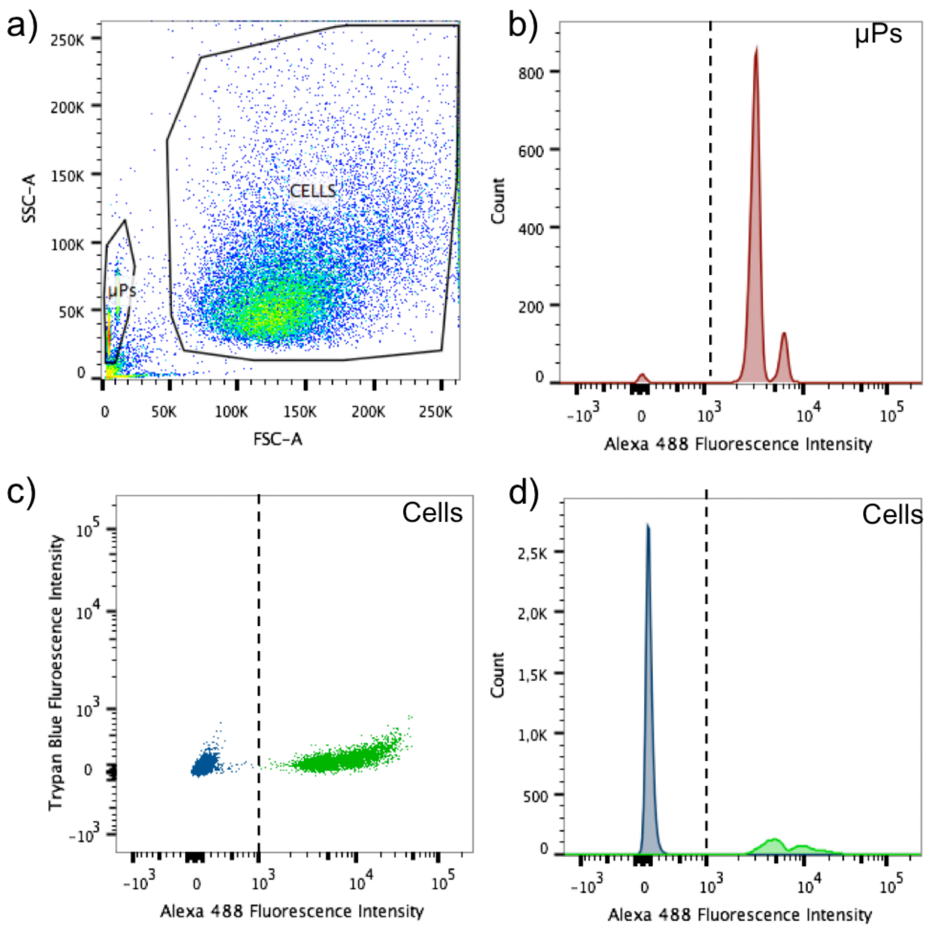

Subset Name	Count	Freq. of Parent
Q4	14838	76,9
Q3	4465	23,1

Subset Name	Count	Freq. of Parent
Q3	4465	23,1
Q4	14838	76,9

Figure S4. Representative flow cytometry analyses, in this case corresponding to MCF-10A cells incubated for 4h with Alexa488-IgG functionalized microparticles, before TB addition. a) Dot plot of the side and forward scatter, where cell and microparticle populations could be easily distinguished. b) Histogram of free Alexa488-IgG microparticles fluorescence. c) Dot plot of cell population showing both Alexa488 and TB fluorescence (in this case there was no red fluorescence signal, as TB was not added to the sample). Different subpopulations of cells were observed, corresponding to cells without microparticles (blue) and cells with microparticles (green). d) Histogram of both subpopulations cells, either with (green) or without (blue) internalized microparticles.
